# Neonatal hypoxic-ischemic encephalopathy diagnosis and treatment: a National Survey in China

**DOI:** 10.1186/s12887-021-02737-6

**Published:** 2021-06-05

**Authors:** Zheng Wang, Peng Zhang, Wenhao Zhou, Shiwen Xia, Wei Zhou, Xiaoyu Zhou, Xiuyong Cheng, Yuan Shi, Zhenlang Lin, Dongli Song, Guoqiang Cheng

**Affiliations:** 1 Department of Neonatology, Children’s Hospital of Fudan University, National Children’s Medical Center, Shanghai, 200032 China; 2 Department of Neonatology, Maternal and Child Health Hospital of Hubei Province, Hubei, 430070 China; 3 Guangzhou women and children’s Medical Center, Guangdong, 510623 China; 4 Children’s Hospital of Nanjing Medical University, Jiangsu, 210093 China; 5 Department of Neonatology, The First Affiliated Hospital of Zhengzhou University, Henan, 450052 China; 6 National Clinical Research Center for Child Health and Disorders, China International Science and Technology Cooperation base of Child development and Critical Disorders, Chongqing Key Laboratory of Pediatrics Children’s Hospital of Chongqing Medical University, Chongqing, 400014 China; 7 The Second Affiliated Hospital and Yuying Children’s Hospital, Wenzhou Medical University, Zhejiang, 325027 China; 8 Department of Pediatrics, Division of Neonatology, Santa Clara Valley Medical Center, San Jose, CA USA; 9 Department of Pediatrics, Stanford University School of Medicine, Palo Alto, CA USA

**Keywords:** Neonatal hypoxic-ischemic encephalopathy, Therapeutic hypothermia, Neuroprotective agent, Long term follow-up

## Abstract

**Background:**

Neonatal hypoxic-ischemic encephalopathy (HIE) affects as many as 100,000 infants each year in China. Therapeutic hypothermia reduces HIE related mortality and long-term neurodevelopmental disabilities. National guidelines for HIE management were published a decade ago. This study aimed to investigate the current status of HIE diagnosis and treatment in China.

**Method:**

This prospective cross-sectional national survey used a questionnaire evaluating practices related to HIE management. Descriptive statistics and Chi-square or Fisher’s exact test were used, and a *p*-value of < 0.05 was considered significant.

**Results:**

The 273 hospitals that completed the survey were located in 31 of the 34 provincial districts in China. Eighty-eight percent of the hospitals were Level III hospitals, and 74% treated 10 or more HIE cases annually. Awareness rates of the national guidelines for HIE diagnosis, HIE treatment, and therapeutic hypothermia protocol were 85, 63, and 78%, respectively. Neurological manifestations and blood gas were used as HIE diagnostic criteria by 96% (263/273) and 68% (186/273) of the hospitals, respectively. Therapeutic hypothermia was used in 54% (147/273) of hospitals. The percentage of general hospitals that implemented therapeutic hypothermia (43%, 71/165) was significantly lower than that in maternity and infant hospitals (67%, 49/73) (*χ*^2^ = 11.752, *p* = 0.001) and children’s hospitals (77%, 27/35) (*χ*^2^ = 13.446, *p* < 0.001). Reasons for not providing therapeutic hypothermia included reduction of HIE cases in recent years (39%), high cost of cooling devices and treatment (31%), lack of training (26%), and safety concerns (4%). Among the hospitals that provided therapeutic hypothermia, 27% (39/147) were in full compliance with the recommended protocol. Eighty-one percent (222/273) of the hospitals treated HIE infants with putative neuroprotective agents alone or in combination with cooling. Ninety-one percent of the hospitals had long-term neurodevelopmental follow-up programs for infants with HIE.

**Conclusions:**

There is significant heterogeneity in HIE diagnosis and treatment in China. Therapeutic hypothermia has not become a standard of care for neonatal HIE nationwide. Unproven agents are widely used for HIE treatment. Nationwide standardization of HIE management and dissemination of therapeutic hypothermia represent the opportunities to reduce mortality and improve long-term neurodevelopmental outcomes of children affected by HIE.

**Supplementary Information:**

The online version contains supplementary material available at 10.1186/s12887-021-02737-6.

## Background

Neonatal hypoxic-ischemic encephalopathy (HIE) is a major cause of mortality and long-term disabilities in children [1–4]. In spite of significant improvements in obstetric and neonatal care, HIE continues to occur in developed countries, with a disproportionately high burden persisting in low- and middle- income countries. HIE occurs in 1 to 3 per 1000 live births in high income countries, and up to 20 per 1000 live births in low and middle-income countries [1, 3–5]. There are 15–18 million live births in China each year, and the incidence of neonatal HIE is estimated to be 3–6 per 1000 live births [6]. Neonatal HIE accounts for 15.2% of mortality under the age of five [7]. Improving survival and long-term neurodevelopmental outcomes of infants with HIE have a significant impact on national and global public health.

Therapeutic hypothermia (TH) for infants with moderate-severe HIE improves survival and decreases long-term disability in infancy and up to mid-childhood [8–16]. TH has become the standard of care in developed countries [17]. To standardize HIE diagnosis and implement evidence-based management of neonatal HIE in China, national guidelines have been published, including “Diagnosis of Hypoxic-ischemic Encephalopathy in Term Infants” [18] (hereinafter referred to as HIE diagnosis guideline), “Guideline of Evidence-based Treatment for Hypoxic-ischemic Encephalopathy in Term Infants” [19] (hereinafter referred to as HIE treatment guideline, Additional file 1), and “Protocol of Hypothermia Treatment for Hypoxic-ischemic Encephalopathy in Neonates” [20] (hereinafter referred to as TH protocol).

Standardization is essential to assure the efficacy and safety of TH in clinical practice and ultimately improve patient outcomes. Since the publications of the national HIE guidelines, many hospitals have implemented TH. However, there has been no report regarding standardization of HIE management and clinical adoption of TH in different types of hospitals throughout China. We conducted a nationwide survey to investigate the current status of neonatal HIE diagnosis, TH, and other HIE treatments, and long-term neurodevelopmental follow-up programs for infants who have survived HIE.

## Methods

### Questionnaire design

The survey was developed by a team of neonatologists at the Children’s Hospital of Fudan University and based on the national HIE guidelines. The survey consisted of 21 questions covering three areas: the characteristics of the survey respondents, HIE diagnosis and treatment, and long-term follow-up (Additional file 2). The questionnaire was presented in the Questionnaire Star format (https://www.wjx.cn/, Hangzhou Oway Medical Technology, Changsha, China).

### Questionnaire affect test

A pre-experiment was performed at the Children’s Hospital of Fudan University to evaluate the questionnaire design. Thirty randomly selected pediatricians took the test. The reliability test demonstrated that the reliability coefficient (Cronbach’s α coefficient) was 0.967 for the total questionnaire, 0.853 for survey respondents’ characteristics, 0.937 for HIE diagnosis and treatment, and 0.89 for follow-up, indicating that the reliability was acceptable. The validity test of the questionnaire was tested by factor analysis. The Kaiser-Meyer-Olkin (KMO) value was 0.844 for the total questionnaire, 0.763 for survey respondents’ characteristics, 0.846 for HIE diagnosis and treatment, and 0.809 for follow-up, indicating that it was suitable for factor analysis.

### Survey method

Between March 12 and April 27, 2019, we conducted a cross-sectional survey through the Neonatal Professional Committee of the Chinese Medical Doctor Association WeChat platform. The questionnaire was distributed to 432 representative neonatologists and pediatricians from general hospitals, maternity and infant hospitals, and children’s hospitals across the country.

### Ethical considerations

This study was approved by the ethics committee of the Children’s Hospital of Fudan University. All respondents participated voluntarily and were informed that the survey data might be used for publication.

### Statistical analysis

The data management and analysis were carried out using the computer software Statistical Package for the Social Sciences (SPSS), version 23.0 (IBM Corp). The descriptive statistics are all presented as numbers and percentages for the categorical variables. Chi-squared or Fisher’s exact tests were applied to find associations or significant differences between the categorical variables, in which *p* < 0.05 was considered statistically significant.

## Results

### Characteristics of survey respondents

**Fig. 1:**
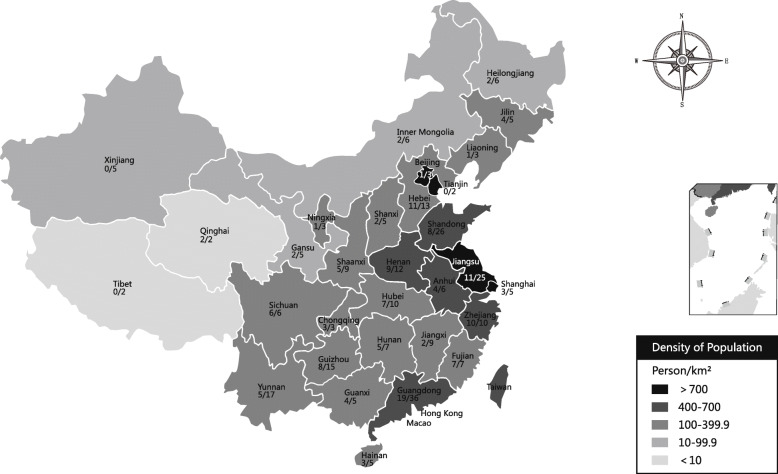
Geographic distribution of survey respondents. A grayscale map of China was divided into five levels depends on population density. The fractions represented the number of hospitals that participated in the survey (denominator) and the number of surveyed hospitals that performed hypothermia treatment (numerator) in each province. The map depicted in Fig. 1 is my own and it was not taken from another source

A total of 432 neonatologists and pediatricians participated in the survey. Sixty-four incomplete responses and 95 redundant responses from the same hospital were removed. Our analysis included 273 (63%) responses, representing 273 hospitals in 127 cities across 31of the 34 provincial districts in China (Fig. 1).

The characteristics of responding physicians and hospitals are summarized in Table 1. Of the 273 responding hospitals, 60% (165/273) were general hospitals, 27% (73/273) were maternity and infant hospitals, and 13% (35/273) were children’s hospitals. Eighty-eight percent were Level III hospitals, accounting for 9.4% Level III hospitals in the nation. Seventy-three percent of the hospitals had 30 or more neonatology beds, and 74% treated 10 or more HIE cases annually.

**Table 1:** Characteristics of the survey respondents

Survey Variables	*N* = 273	
Profession, No. (%)		
Neonatologist	196	(72)
Pediatrician	77	(28)
Professional title, No. (%)		
Senior	203	(74)
Intermediate	60	(22)
Junior	10	(4)
Type of hospital, No. (%)		
General hospital	165	(60)
Maternity and infant hospital	73	(27)
Children’s hospital	35	(13)
Level of hospital, No. (%)		
III	239	(88)
II	33	(12)
I	1	(0.4)
NICU beds, No. (%)		
>200	9	(3)
151–200	15	(5)
101–150	26	(10)
61–100	60	(22)
30–60	100	(37)
<30	63	(23)
HIE cases treated annually, No. (%)		
>50	47	(17)
40–50	10	(4)
30–40	26	(10)
20–30	53	(19)
10–20	66	(24)
<10	71	(26)

*Abbreviation*: *NICU* Neonatal intensive care unit, *HIE* Hypoxic-ischemic encephalopathy

### Diagnosis of HIE

The HIE diagnosis criteria used in the 273 surveyed hospitals are shown in Table 2. Neurological manifestations and umbilical cord or infant arterial blood gases were used as HIE diagnostic indicators by 96% (263/273) and 68% (186/273) of hospitals, respectively. Electroencephalography (EEG) and/or amplitude-integrated electroencephalography (aEEG) were used by 56% (152/273) of the hospitals, and magnetic resonance imaging (MRI) was used by 73% (200/273) of the hospitals. There were no statistically significant differences in utilizing different diagnostic indicators among the three types of hospitals.

**Table 2:** HIE Diagnosis, Treatments and Follow-up

Survey Variables, ***n*** (%)	Total hospital	General hospital	Maternity and infant hospital	Children’s hospital	***P***-value
	***n*** = 273	***n*** = 165	***n*** = 73	***n*** = 35	
Which indicator(s) do you use for diagnosing HIE?					0.516
Neurological manifestations	263 (96.3)	157 (95.2)	71 (97.3)	35 (100.0)	
Arterial blood gas	186 (68.1)	110 (66.7)	49 (67.1)	27 (77.1)	
Abnormal EEG/aEEG	152 (55.7)	79 (47.9)	44 (60.3)	29 (82.9)	
Abnormal MRI	200 (73.3)	126 (76.4)	43 (58.9)	31 (88.6)	
CT low density shadow	79 (28.9)	44 (26.7)	25 (34.2)	10 (28.6)	
Blood neuroenzymatic changes	86 (31.5)	52 (31.5)	18 (24.7)	16 (45.7)	
Does your hospital provide TH for infants with HIE?					< 0.001
Yes	147 (53.8)	71 (43.0)	49 (67.1)	27 (77.1)	
Do you use any of the following agents to treat HIE?					0.462
Cytidine diphosphate choline	57 (20.9)	41 (24.8)	8 (11.0)	8 (22.9)	
Rat nerve growth factor	70 (25.6)	46 (27.9)	11 (15.1)	13 (37.1)	
Ganglioside	179 (65.6)	111 (67.3)	45 (61.6)	23 (65.7)	
Erythropoietin	37 (13.6)	21 (12.7)	11 (15.1)	5 (14.3)	
Cerebrolysin	24 (8.8)	17 (10.3)	5 (6.8)	2 (5.7)	
Does your hospital have a long-term follow-up service for HIE children?					0.69
Yes	248 (90.8)	149 (90.3)	68 (93.2)	31 (88.6)	
Up to how old are the HIE children followed up?					0.715
1 year old	41 (15.0)	28 (17.0)	9 (12.3)	4 (11.4)	
2 years old	86 (31.5)	48 (29.1)	26 (35.6)	10 (28.6)	
Preschool age	148 (54.2)	89 (53.9)	38 (52.1)	21 (60.0)	
Which neurodevelopmental assessment scale(s) is used in your follow-up clinic?					0.106
Bayley	140 (51.3)	76 (46.1)	45 (61.6)	19 (54.3)	
CDCC	69 (25.3)	45 (27.3)	18 (24.7)	6 (17.1)	
Other standardized tests^a^	30 (11.0)	22 (13.3)	2 (2.7)	6 (17.1)	
None of above	34 (12.5)	22 (13.3)	8 (11.0)	4 (11.4)	

*Abbreviation*: *HIE* Hypoxic-ischemic encephalopathy, *TH* Therapeutic hypothermia, *EEG* Electroencephalography, *aEEG* amplitude-integrated electroencephalography, *MRI* Magnetic resonance imaging, *CT* Computerized tomography, *GMs* General movements, *NBNA* Neonatal behavioral neurological assessment, *DDST* Denver developmental screening test

^a^ Include Gesell, GMs, NBNA, Wechsler intelligence scale, and DDST

### Treatment for HIE

Fifty-four percent (147/273) of the surveyed institutions provided TH, including 143 Level III (143/239, 60%) and 4 (4/33, 12%) Level II hospitals. Ninety-seven percent (143/147) of the cooling hospitals are level III hospitals. The percentage of general hospitals that provided TH (43%, 71/165) was significantly lower than that in maternity and infant hospitals (67%, 49/73) (*χ*^2^ = 11.752, *p* = 0.001) and children’s hospitals (77%, 27/35) (*χ*^2^ = 13.446, *p* **<** 0.001) (Table 2).

Several factors were identified as reasons for not offering TH (Table 3), including reduction of HIE cases in recent years (39%), high cost for cooling devices and TH treatment (31%), lack of training in TH (26%), and concerns about the safety of TH in newborns (4%). There were no statistically significant differences in these reasons among the three types of hospitals.

**Table 3:** Reasons for Not Providing Therapeutic Hypothermia

Reasons, ***n*** (%)	Total hospital	General hospital	Maternity and infant hospital	Children’s hospital
	***n*** = 126	***n*** = 94	***n*** = 24	***n*** = 8
Reduction of HIE cases	49 (38.9)	34 (36.2)	12 (50.0)	3 (37.5)
High cost of cooling devices	39 (31.0)	29 (30.9)	7 (29.2)	3 (37.5)
Lack of training in TH	33 (26.2)	26 (27.7)	5 (20.8)	2 (25.0)
Concerns about the safety of TH	5 (4.0)	5 (5.3)	0 (0.0)	0 (0.0)

*Abbreviation*: *HIE* Hypoxic-ischemic encephalopathy, *TH* Therapeutic hypothermia

Of the hospitals that provided TH, 86% (126/147) used a servo-controlled cooling device, including whole-body cooling (69%) or selective head cooling (31%). Ten percent use simple cooling methods (e.g., cold water bags or fans), and 4 % used passive hypothermia (no added heating source). Overall, 27% (39/147) of the cooling hospitals were fully compliant with the national TH treatment guideline and protocol; 31% followed TH patient selection criteria; 93% initiated cooling at < 6 h of life, 95% targeted a cooling temperature at 33–34 °C, and 90% cooled for 72 h. There is no statistically significant difference in compliance among the three types of hospitals (Table 2).

Eighty-one percent (222/273) of surveyed institutions treated HIE infants with putative neuroprotective agents (Table 2). Of the five agents, ganglioside was most commonly used. Eighteen percent of hospitals used one agent alone, 16% used two or more agents, 29% used one agent combined with TH, and 19% used two or more agents combined with TH. There is no statistically significant difference in use of the putative neuroprotective agents among the three types of hospitals (Table 2).

### Long-term neurodevelopmental follow-up

Of the 273 surveyed hospitals, 91% (248) have long-term neurodevelopmental follow-up programs for HIE infants. The Bayley III scale was used in 51% of the follow-up assessments.

### Impact of National Guidelines on HIE diagnosis and treatment

Of the 273 respondents, the awareness rates of the national guidelines for HIE diagnosis, HIE treatment, and TH protocol were 85, 63, and 78%, respectively. There were no statistically significant differences in awareness of the guidelines between the three different types of hospitals. The respondents who were aware of the HIE diagnosis guidelines were more likely to use blood gas and EEG/aEEG as indicators for HIE diagnosis than those who were unaware of the guideline (Table 4). The rates of TH adoption, TH protocol compliance, and use of putative neuroprotective agents were similar in the hospitals that were aware vs. unaware of the HIE treatment guidelines (Table 4).

**Table 4:** Impact of Awareness of HIE Diagnosis and Treatment Guidelines

**HIE diagnostic indicators,** ***n*** **(%)**	**HIE diagnosis**		***P*-value**
	**Aware** ***n*** **= 232 (85)**	**Unaware** ***n*** **= 41**	
Neurological manifestations	222 (95.7)	41 (100.0)	0.366
Arterial Blood gas	167 (72.0)	19 (46.3)	0.001
Abnormal EEG/aEEG	136 (58.6)	16 (39.0)	0.02
Abnormal MRI	174 (75.0)	26 (63.4)	0.122
CT low density shadow	66 (28.4)	13 (31.7)	0.671
Blood neuroenzymatic changes	75 (32.3)	11 (26.8)	0.485
	**HIE Treatment**		
	**Aware** ***n*** **= 171 (63)**	**Unaware** ***n*** **= 102**	
**Rate of TH,** ***n*** **(%)**	98 (57.3)	49 (48.0)	0.137
**Neuroprotection agents,** ***n*** **(%)**	133 (77.8)	89 (87.3)	0.052
	**TH Protocol**		
	**Aware** ***n*** **= 129 (88)**	**Unaware** ***n*** **= 18**	
**Rate of TH compliance,** ***n*** **(%)**	37 (28.7)	2 (11.1)	0.195

*Abbreviation*: *HIE* Hypoxic-ischemic encephalopathy, *EEG* Electroencephalography, *aEEG* amplitude-integrated electroencephalography, *MRI* Magnetic resonance imaging, *CT* Computerized tomography, *TH* Therapeutic hypothermia

## Discussion

This is the first national survey of neonatal HIE diagnosis and treatment conducted in China. The respondents were mainly senior pediatricians and neonatologists working in Level III hospitals across the country. Thus, findings from our survey provide a good representation of the current status of neonatal HIE diagnosis and treatment in China.

The 2005 updated Chinese national HIE diagnosis guideline [18] requires meeting all of the following four criteria: (1) history of perinatal hypoxic-ischemic events and evidence of fetal distress, (2) Apgar scores at 1 and 5 min of </= 3 and </=5, respectively, and or arterial blood gas pH </= 7, (3) evidence of encephalopathy, and (4) exclusion of other causes for encephalopathy. Our survey found that 96% of respondents used clinical manifestations of encephalopathy as HIE diagnostic criteria, but only 68% used arterial blood analysis as a diagnostic indicator. Physicians who were aware of the HIE diagnostic criteria were more likely to use arterial blood gas. This demonstrates that more education is needed to emphasize the value of cord blood gas analysis in providing essential evidence of acute hypoxic-ischemic insults to the fetus during labor and delivery. Additionally, placing designated blood gas analyzers may facilitate the utilization of blood gas by labor and delivery departments that have thousands of deliveries each year. The HIE diagnostic guideline also recommends using EEG/aEEG and brain imaging (preferably MRI when available) to obtain information on brain injury and prognosis. In our survey, only 56% of hospitals utilized EEG/aEEG, and 73% used brain MRI, which represents an significant opportunity to improve HIE diagnosis and prognosis [21–23].

International multicenter randomized clinical trials (RCT) and subsequent meta-analysis have demonstrated that TH is effective and safe for treating neonates with moderate and severe HIE [8–13]. In developed countries, much effort has been devoted to disseminating and standardizing TH in clinical practice [24–29], leading to TH becoming the standard of care in clinical practice. The implementation of TH in developing countries faces multiple challenges [30–32]. While data from low-resource settings is limited, a recent systemic review showed that hypothermia efficacy was not associated with countries’ income levels [33]. TH was recommended by the Chinese national guidelines a decade ago, it was implemented only in 54% of the surveyed hospitals, and only 60% of the Level III hospitals that routinely treat critically ill neonates offered the therapy. Awareness of the HIE treatment guideline and TH protocol was only 63 and 78%, respectively. Furthermore, awareness of the guidelines was not associated with a higher rate of TH.

It is worthwhile noticing that the TH rate in general hospitals (43%) was significantly lower than that in maternity and infant hospitals (67%) and children’s hospitals (77%). This difference demonstrates that hospitals specializing in women’s and children’s services place a higher priority on implementing novel neonatal therapies (like TH) and support such efforts with necessary funding and resources.

Thirty-nine percent of the respondents listed reduction in HIE cases in recent years as a reason for not providing TH. In the past decades, China has made remarkable progress in maternal and child health. Between 1990 and 2018, the national neonatal mortality rate has decreased from 30 to 4 deaths per 1000 live births, and the under-5 mortality rate decreased from 54 to 8.5 per 1000 live births [34]. National birth-asphyxia-related infant mortality has been reduced significantly since the launch of China’s Neonatal Resuscitation Program (NRP) in 2004 [35]. Despite this progress, HIE affects as many as 100,000 newborns each year in China and remains the leading national cause of mortality and morbidities in children under-5 years of age [7]. In our survey, 74% of hospitals treated more than 10 cases of HIE annually, but just 54% of the hospitals provided TH, the only proven therapy for neonatal HIE. Given the high rates of mortality and morbidity associated with HIE, every affected newborn should have the opportunity to benefit from TH treatment.

Thirty percent of survey respondents expressed a lack of training in TH and concerns about the safety of hypothermia treatment. This finding, together with the observation of low awareness of the HIE guidelines, highlights a significant need for a national education program like NRP, that provides organized education and training in TH throughout the county. Another 31% of respondents cited the high cost of cooling devices and treatment. While hospital funding in China has improved dramatically in recent years, this has brought up the need for more financial investment in lifesaving therapies in neonatal critical care. In addition, financial reimbursement to the hospitals for providing TH therapy remains to be established.

The Chinese TH protocol [20] follows the published patient selection criteria and cooling methods used in multicenter RCTs [8–10]. Our survey showed that only 27% of the cooling hospitals were in full compliance with published TH protocol. Only 31% followed the recommended patient selection criteria, which reflects the lack of standardization in HIE diagnosis. Compliance issues were also found in cooling procedures, including the timing of TH initiation, cooling temperature, and cooling duration. Similar practice variations have been reported by a recent study in Brazil [32]. Standardization in the health care setting has been shown to improve safety and patient outcomes [36]. While current recommended TH protocols are being optimized through more clinical trials [37, 38], standardization is a crucial component of TH dissemination to assure its efficacy and safety in clinical practice.

Despite TH therapy, up to 55% of children with HIE die or suffer severe long-term neurological abnormalities [13], emphasizing the need for further research for new treatments. Multicenter RCTs are investigation the neuroprotective effects of erythropoietin, allopurinol, xenon, melatonin, and topiramate when administrated alone or as adjuvants to TH [39–43]. Our survey found that 81% of the hospitals used one or more putative neuroprotective agents, including erythropoietin, ganglioside, rat nerve growth factor, citicoline, and cerebrolysin [44–48]. Clinical trials conducted in China and meta-analyses of these studies showed that ganglioside, the most commonly used agent in our survey, was safe and effective in reducing neurological sequelae of HIE [45]. However, the trials had significant heterogeneities in study design, patient population selection, and outcome evaluation. Most of the studies lack data on long-term follow-up. Rat nerve growth factor, citicoline, and cerebrolysin were mainly evaluated by pre-clinical investigations and limited clinical studies [46–48]. Their neuroprotective effect remains to be demonstrated by multicenter RCTs.

Long-term neurodevelopmental follow-up is an essential component of care for children affected by HIE, which allows early identification of and intervention for abnormal development. Our survey showed that 91% of hospitals had a long-term follow-up program; more than half of them used the Bayley III scale. Nationwide standardization in timing and method of neurodevelopmental assessment remains to be established.

This study has several limitations. We were not able to provide the overall rate of HIE, percentage of HIE infants who received TH, and complications of TH because many of the surveyed hospitals did not have a database for their HIE cases. This is due to the fact that majority of the survey hospitals were lack of database to track HIE infants. Establishing a national neonatal registry is essential for providing epidemiological data on neonatal HIE incidence, treatments, and outcomes [24]. This survey mainly reflects the knowledge of providers who practice in Level III hospitals. However, many babies are delivered in Level I and II hospitals. Future surveys should include providers in Level I and II hospitals to assess their abilities to timely identify at-risk newborns, stabilize, and transfer these infants to cooling centers [22]. as many of the survey hospitals were lack of a database tracking HIE cases.

## Conclusion

There is significant heterogeneity in HIE diagnosis and management in China. TH has been implemented in many hospitals but has not become a standard of care for neonatal HIE. Our survey identified barriers and improvement opportunities to disseminate evidence-based clinical practice. The recent establishment of the China Neonatal Neurointensive Care Alliance [49] will facilitate nationwide education, standardize HIE diagnosis and treatment, organize multicenter RCTs, and advocate funding for novel therapies for neonatal HIE.

## Supplementary Information

**Additional file 1.** Guidelines for Evidence-Based of Hypoxic-Ischemic Encephalopathy in Full-Term Infants (2011-Simplified Edition).

**Additional file 2.** Survey questions.

## Data Availability

The datasets used and/or analyzed during the current study are available from the corresponding author on reasonable request.
