# Supplementary material for: Neonatal hypoxic-ischemic encephalopathy diagnosis and treatment: a National Survey in China
Source: BMC Pediatr. 2021 Jun 5;21:261. doi: 10.1186/s12887-021-02737-6 (PMC8178820; doi:10.1186/s12887-021-02737-6)
Supplement: Supplementary file 1 — Additional file 1. Guidelines for Evidence-Based of Hypoxic-Ischemic Encephalopathy in Full-Term Infants (2011-Simplified Edition). [file 12887_2021_2737_MOESM1_ESM.doc]

Appendix A

**Guidelines for Evidence-Based of Hypoxic-Ischemic Encephalopathy in Full-Term Infants (2011-Simplified Edition).**

Key Laboratory of Neonatal Diseases, Ministry of Health, Children’s Hospital of Fudan University, Editorial Office of Chinese Journal of Evidence Based Pediatrics, GRADE Working Group China Center. DOI:10.3969/j.issn.1673-5501.2011.05.004

Recommendations of supportive and specific neuroprotective therapy for full-term infants with HIE

| **Recommendations** | **Explanation** | **Source of Evidence** |
| --- | --- | --- |
| **Support symptomatic treatment** | | |
| Maintaining adequate ventilation and oxygenation is recommended (1D) | Both hypoxemia and severe hypercapnia can impair the autonomic regulation of cerebral blood flow and lead to passive cerebral circulation | Classic monographs ^1)^ |
| Maintaining appropriate cerebral perfusion is recommended (1D) | Because of the pressure-reactive cerebral circulation of HIE, any slight fluctuation in blood pressure can aggravate brain damage | Classic monographs ^1)^ |
| Maintaining appropriate blood glucose level is recommended (1D) | Both hypoglycemia and hyperglycemia are not beneficial for neonatal HIE | Classic monographs ^1)^ |
| Appropriate restriction of fluid intake is recommended to prevent cerebral edema (1D); Routine use of mannitol for the prevention of cerebral edema is not recommended (2D); Hormone is not recommended to reduce cerebral edema (2D) | Excessive fluid supply in HIE children may increase the amount of water in the brain tissue and aggravate brain damage, but not at the expense of normal blood pressure and stable internal environment | Classic monographs ^1)^ |
| Phenobarbital is recommended as the first-line therapy for the control of convulsions (1D), and phenobarbital is not recommended for the prevention of convulsions in full-term infants with HIE (2C). | Convulsion can aggravate brain injury, but prophylactic phenobarbital does not reduce the mortality and severe disability | One Meta- analysis (10 RCTs) |
| **Special neuroprotective therapy** | | |
| Therapeutic hypothermia is recommended for the treatment of moderate and severe in full-term infants with HIE (1A). | It can significantly reduce the rate of mortality (RR=0.58, 95%CI: 0.45-0.75), the rate of morbidity (RR= 0.76, 95% CI: 0.68-0.84) and severe long term disability (RR= 0.76, 95%CI: 0.68-0.84) of full-term infants with HIE. | One updated meta-analysis (18 RCTs) |
| Hyperbaric oxygen is not recommended for full-term infants with HIE (2D). | The efficacy and safety of hyperbaric oxygen treatment for full-term infants with HIE have not been thoroughly evaluated by multi-center RCTs. | One updated meta-analysis (11 RCTs) |
| Erythropoietin is not recommended for HIE in full-term infants (2C). | The results of a single center study were insufficient to confirm the neuroprotective effect. | One RCT |
| Human neural stem cell transplantation is not recommended for HIE in full-term infants (2D). | Evidence from only two case reports | Case reports |
| Magnesium sulfate is not recommended for HIE in full-term infants (2D). | This treatment for severe neonatal asphyxia did not reduce the mortality rate (RR = 1). | One RCT |
| Allopurinol is not recommended for HIE in full-term infants (2C). | t did not reduce the mortality of full-term infants with HIE (RR=0.92, 95%CI: 0.59-1.45) and the incidence of convulsion (RR=0.93, 95%CI: 0.75-1.16). | One Meta- analysis (3 RCTs) |
| Naloxone is not recommended for full-term infants with HIE (2C). | Although one meta-analysis showed good outcome, there were serious limitations in the original studies. | One Meta -analysis (20 RCTs) |
| It is not recommended to treat full-term infants with HIE with cyticholine, brain activating hormone and fructose 1, 6-diphosphate (2D). | 13 votes support, 27 votes against | Delphi method |
| Ganglioside is not recommended for HIE in full-term infants (2D). | 13 votes support, 27 votes against | Delphi method |
| Basic fibroblast growth factor and nerve growth factor are not recommended for HIE in full-term infants (2D). | 5 votes support, 35 votes against | Delphi method |
| Ibuprofen and indomethacin are not recommended for HIE in full-term infants (2D). | 20 votes support, 20 votes against | Delphi method |
| Nifedipine, nimodipine, and Sichuan bow oxazine are not recommended for the treatment of HIE in full-term infants (1D). | 21 votes support, 19 votes against | Delphi method |
| Anisodamine and anisodamine are not recommended for the treatment of HIE in full-term infants (1D). | 21 votes support, 18 votes against, 1 invalid vote | Delphi method |

Note: 1) The evidence of support symptomatic treatment in this guide comes from four classic monographs: Volpe JJ, *Neurology of the newborn*, Version 5; Taeusch HW; *Avery’s diseases of the newborn*, Version 8; Xiaomei Shao, Hongmao Ye, Xiaoshan Qiu, *Practice of neonatology*, Version 4; Yukun Han, Yujia Yang, Xiaomei Shao, *Neonatal hypoxic-ischemic encephalopathy*, Version 2.
