# Supplementary material for: Neonatal hypoxic-ischemic encephalopathy diagnosis and treatment: a National Survey in China
Source: BMC Pediatr. 2021 Jun 5;21:261. doi: 10.1186/s12887-021-02737-6 (PMC8178820; doi:10.1186/s12887-021-02737-6)
Supplement: Supplementary file 2 — Additional file 2. Survey questions. [file 12887_2021_2737_MOESM2_ESM.doc]

**Appendix B Survey questions**

1. **What is your specialty?**
2. Neonatologist
3. General pediatrician
4. Neonatal nurse
5. Neurodevelopmental pediatrician
6. Other (specify)

1. **What is your professional level?**
2. Senior
3. Intermediate
4. Junior
5. Other (specify)

1. **What type of hospital where you work?**
2. General hospital
3. Maternity and infant hospital
4. Children’s hospital
5. Other (specify)

1. **What level of the hospital where you work?**
2. III level
3. II level
4. I level

1. **How many beds in your neonatal unit?**
2. ＜30
3. 30-60
4. 61-100
5. 101-150
6. 151-200
7. ＞200

1. **How many cases of neonates hypoxic-ischemic encephalopathy (HIE) are treated in your hospital each year?**
2. ＜10
3. 10-20
4. 21-30
5. 31-40
6. 41-50
7. ＞50

1. **Are you aware of the experts’ consensus of HIE diagnosis in China?**
2. Yes
3. No
4. I do not know

1. **Which indicator(s) do you use for diagnosing HIE?**
2. Neurological manifestations
3. Arterial blood gas
4. EEG
5. Brain MRI
6. Brain CT
7. Blood tests of brain enzymatic

1. **Are you aware of the evidence-based HIE guidelines in China?**
2. Yes
3. No
4. I do not know

1. **Are you aware of the Therapeutic Hypothermia Protocol in China?**
2. Yes
3. No
4. I do not know

1. **Does your hospital provide therapeutic hypothermia for infants with HIE?**
2. Yes
3. No
4. I do not know

1. **What is the method of therapeutic hypothermia used in your hospital?**
2. Cooling device
3. low-cost devices
4. Passive hypothermia by simply turning off the heating
5. I do not know

1. **What type of cooling device for therapeutic hypothermia is used in your hospital?**
2. Selective head cooling
3. Whole-body cooling
4. I do not know

1. **What is the time window for initiating therapeutic hypothermia?**
2. ＜6 hours
3. ＜12 hours
4. ＜24 hours
5. I do not know

1. **What is the target temperature for therapeutic hypothermia?**
2. ＜33 ℃
3. 33-33.5 ℃
4. 33.6-34 ℃
5. I do not know

1. **What is the duration of therapeutic hypothermia?**
2. 48 hours
3. 72 hours
4. 96 hours
5. I do not know

1. Do you use any of the following agents to treat HIE?
2. Cytidine diphosphate choline
3. Rat nerve growth factor
4. Ganglioside
5. Erythropoietin
6. Cerebrolysin

1. **Does your hospital have a long-term follow-up service for HIE children?**
2. Yes
3. No

1. **Up to how old are the HIE children followed up?**
2. 1 year old
3. 2 years old
4. Preschool age
5. **Which neurodevelopmental assessment scale(s) are used in your follow-up clinic?**
6. Bayley
7. CDCC
8. Other (specify)
9. None

1. **Which department in your hospital performs neurodevelopmental assessments for infants with HIE?**
2. Child health care departments
3. Child rehabilitation
4. Neonatology
5. Neurology
